# Serum Hepcidin Levels in Breast Cancer Patients: Investigating the Correlation to Tumor Stage—A Cross‐Sectional Study

**DOI:** 10.1002/hsr2.71148

**Published:** 2025-08-10

**Authors:** Zein Al‐Abideen Douba, Rama Ibrahim

**Affiliations:** ^1^ Department of Biochemistry and Microbiology, Faculty of Pharmacy Tishreen University Lattakia Syria

**Keywords:** anemia, biomarkers, breast cancer, CA 15‐3, hepcidin

## Abstract

**Background and Aims:**

Breast cancer is one of the most common types of cancer worldwide. Hepcidin is a liver‐produced hormone playing a key role in the regulation of iron levels in the body. Studies have shown that hepcidin is upregulated in many diseases and inflammatory conditions, including cancers. This study paper aims to assess serum hepcidin in patients with breast cancer and to study its correlation with tumor stage.

**Methods:**

A total of 39 breast cancer women and 25 healthy controls were included in the study. Serum hepcidin levels were measured using the ELISA method. Other tumor‐related parameters including Cancer Antigen 15‐3 (CA 15‐3), Iron (Fe), C‐Reactive Protein (CRP) and the complete blood count (CBC) were also assessed.

**Results:**

This study showed Furthermore, this study found a positive correlation a significant increase in hepcidin levels in breast cancer patients compared to healthy controls (*M* = 5.57, SD = 4.87), *t*(62) = 6.89, *p* < 0.001, Cohen's *d* = 1.75. Furthermore, serum hepcidin showed a strong positive correlation with CA15‐3 (*r* = 0.62, *p* < 0.001), CRP (*r* = 0.58, *p* < 0.001), and tumor stage (*η*² = 0.47, *p* < 0.001), indicating its potential as a prognostic marker.

**Conclusion:**

These findings suggest that serum hepcidin may serve as a potential biomarker to monitor disease progression in breast cancer patients. CRP could be a positive‐regulator for hepcidin in breast cancer.

## 1 Introduction

Breast cancer has replaced lung cancer as the most commonly diagnosed cancer globally, and today accounts for 1 in 8 cancer diagnoses and a total of 2.3 million new cases in both sexes combined [1]. This cancer represents a quarter of all cancer cases in females, and was by far the most common cancer in women in 2020 [2]. An estimated 685,000 women died from breast cancer in 2020, amounting to 16% (one in six) of cancer deaths among women [3, 4]. According to World Health Organization statistics for the year 2020, breast cancer comes first among women in Syria with an incidence rate of 37.5%, followed by colorectal cancer 7.3% [2]. The few available studies and reports indicate that the average age of women diagnosed with breast cancer in Syria is 49 years, a decade younger than the average age in developed countries [5, 6].

Due to its high prevalence, breast cancer remains a major health concern worldwide, with an urgent need to understand its underlying mechanisms for more effective treatment strategies [7]. While advances in screening and treatment have improved outcomes, there is still a need for better biomarkers to monitor disease progression and response to treatment [8, 9, 10]. Hepcidin is a protein that is produced in the liver [11]. It's considered a master regulator of iron entry into the circulation in mammals [11, 12]. Hepcidin functions to block the export and release of cellular iron into the plasma from macrophages, as well as the recycling of iron from consumed red blood cells [13, 14, 15]. In addition, it inhibits dietary iron absorption through downregulation of ferroportin, a transmembrane iron transporter [15]. This multitude of hepcidin functions makes it a major regulator of iron available in plasma and cellular iron stores [16]. Serum hepcidin levels are affected by certain physiological conditions, such as erythropoietic activity and iron status [11, 17], as well as pathologies, including chronic inflammation and malignancies, in which there is defective iron utilization and a diminished erythropoietin response [18, 19]. When hepcidin levels are abnormally high, blood iron decreases because iron becomes trapped within macrophages and liver cells, and iron absorption from the intestine is reduced [20, 21]. This usually leads to anemia, frequently seen in inflammatory conditions and cancers [20, 22]. Breast cancer is closely related to altered iron metabolism [22]. While the exact pathophysiological link is still undetermined, it is generally accepted that breast cancer has variable iron requirements due to rapid proliferation and high energy demands [23]. Significant expression of hepcidin has been reported in breast cancer cell lines, hypothesizing that these cells thus have the potential to maintain high iron levels, thereby avoiding the cytotoxic effects of iron deprivation [24, 25, 26]. Consistent with this, higher hepcidin levels have been observed in breast cancer patients compared to healthy individuals, suggesting a possible relationship between hepcidin and breast cancer development [25]. Furthermore, high hepcidin level has also been linked to poor prognosis in breast cancer patients, as it was associated with increased tumor size, higher grade tumors, and a more aggressive disease course [27, 28]. However, the role of hepcidin in breast cancer pathogenesis has not been confirmed, with some studies reporting no significant differences [29, 30, 31]. Due to the conflicting results on this topic, this study aimed to fill the gap regarding the significance of serum hepcidin in breast cancer and to investigate its relationship with cancer stage and patient's susceptibility to recurrence. Unlike most reference studies, which assessed hepcidin levels in breast cancer patients a year and a half or more after treatment to compare relapsing and non‐relapsing patients‐demonstrating elevated hepcidin levels in those who recurrence, This study measured hepcidin levels at diagnosis, before any treatment was initiated, to predict which patients might recurrence based on hepcidin values. This study also assessed the correlation between hepcidin levels and a commonly used breast cancer biomarker, the cancer antigen 15‐3 (CA15‐3), and one of the potential regulators of hepcidin in cancers, the C‐reactive protein (CRP). The results of this study will provide valuable insights on the use of hepcidin as a potential diagnostic and prognostic biomarker for breast cancer.

## 2 Materials and Methods

### 2.1 Study Design and Participants

The study included 64 women: 39 women with breast cancer at different stages (13 patients at each of stage I, II, and III) and 25 healthy controls. Blood samples were drawn from the patients upon admission to the oncology department of Tishreen University Hospital during the period from March 2022 to September 2022, before receiving any chemotherapy.

**Inclusion criteria:**

- Newly diagnosed breast cancer patients before receiving any treatment.
- Patients diagnosed with stage I, II, or III breast cancer.
- Patients with complete medical records and consent for participation.

**Exclusion criteria:**

- Patients with metastatic breast cancer.
- Patients who had already started chemotherapy before sample collection.
- Patients with acute liver disease or acute kidney disease.
- Hemolyzed blood samples, which could interfere with accurate biochemical measurements.

Tumor stage was determined based on histopathological examination of tumor tissue. All patients with acute liver disease, acute kidney disease, hemolyzed blood samples, those who had begun chemotherapy, and patients with metastases were excluded from the study. Serum hepcidin was analyzed using the American‐made ELISA kit from (R&D Systems Inc., a Bio‐Techne brand). Iron and CRP tests were performed using a BS‐350 device from the Chinese company Mindray. Red Blood Cells (RBC), White Blood Cells (WBC), Hemoglobin (Hb) and the Mean Corpuscular Volume (MCV) were measured with the Sysmex XT‐1800i analyzer. CA15‐3 analysis was performed using the automated immunoassay analyzer 360.

The study protocol was reviewed and approved by the Scientific Research Ethics Committee at Tishreen University and Tishreen University Hospital. The approval process was facilitated by the Office of External Relations at Tishreen University, under the supervision of Dr. Sawsan Ghazal. Written informed consent was obtained from all participants before enrollment. Patients were informed about the purpose of the study, the procedures involved, potential risks, and their right to withdraw at any time without consequences. The study was conducted in compliance with the Declaration of Helsinki and all applicable ethical standards.

### 2.2 Statistical Analysis

Statistical analyzes were conducted using the Statistical Package for the Social Sciences (SPSS), version 26. The Shapiro‐Wilk test was performed to check the normal distribution. Categorical variables were expressed as number (*n*) and percentage (%) and compared using the Chi‐square test. Continuous variables were expressed as mean and standard deviation (SD), and comparison between groups was performed as follows:The Independent sample *t*‐test or the nonparametric Mann– Whitney U test were used for comparisons between two groups according to variable distribution, while the Oneway ANOVA test or the Kruskal–Wallis test were performed for comparisons between several groups according to variable distribution.

The receiver operation characteristic curve (ROC Curve) was used to analyze the optimal cut‐off value of hepcidin for predicting recurrence in breast cancer patients. Kaplan–Meier curves were constructed to trace the progression free survival (PFS) and the overall survival (OS) among breast cancer patients. Results were considered statistically significant when *p* < 0.05.

Receiver operating characteristic (ROC) curve analysis was performed to evaluate the diagnostic performance of CRP and hepcidin in distinguishing breast cancer patients from controls. The area under the curve (AUC), cut‐off values, sensitivity, and specificity were determined using the Youden Index. Statistical analyses were conducted using SPSS version 26 (IBM Corp., USA).

All statistical analyses were performed using SPSS version 26 (IBM Corp., USA). Normality of data distribution was assessed using the Shapiro‐Wilk test. Categorical variables were expressed as frequencies and percentages and compared using the Chi‐square test. Continuous variables were expressed as means ± standard deviations (SD) or medians with interquartile ranges (IQRs), depending on normality. Comparisons between two groups were performed using the Independent Sample *t*‐test for normally distributed variables or the Mann–Whitney U test for non‐normally distributed variables.

One‐way ANOVA with post‐hoc Tukey's test was used for multiple group comparisons when data were normally distributed, while the Kruskal‐Wallis test followed by Dunn's post‐hoc test was applied for non‐parametric data.

Effect sizes were reported using Cohen's d for *t*‐tests, eta squared (*η*²) for ANOVA, and Cliff's delta for nonparametric tests where applicable. A *p* < 0.05 was considered statistically significant

Effect sizes were interpreted as follows: Cohen's *d*: Small (0.2), Medium (0.5), Large (0.8), Eta squared (*η*²): Small (0.01), Medium (0.06), Large (0.14), Pearson's *r*: Small (0.1), Medium (0.3), Large (0.5), Statistical significance was set at *p* < 0.05.

## 3 Results

### 3.1 Baseline Characteristics of Study Participants

The study sample included 64 women, divided into two groups as follows: the patient group, which included 39 women diagnosed with breast cancer; ages ranged between 37 and 76 years with a mean of 57 ± 8.9 years, and the control group, which composed of 25 healthy women, ages ranged from 30 to 64 years with a mean of 52.3 ± 10.9 years. The study follows CONSORT guidelines for transparent reporting. Patients were classified according to tumor stage, and all analyses were performed according to the predefined statistical plan. The inclusion and exclusion criteria were strictly followed to ensure the reliability of the findings Breast cancer patients were selected equally at all stages, and all were followed for 18 months. Of the 39 patients, 15 patients (38.5%) recurrenced during the follow‐up period. Among them, 10 patients (25.6%) died. Among the patients, 6 were Grade 1 (15.38%), 14 were Grade 2 (35.89%), and 19 were Grade 3 (48.71%). In terms of receptor status, only 2 patients (5.1%) were hormone receptor‐negative/HER2‐positive, 15 patients (38.5%) were hormone receptor‐positive/HER2‐negative, 15 patients (38.5%) were triple‐positive, and 7 patients (17.9%) were triple‐negative (Table 1).

**Table 1:** Baseline characteristics.

Variables		Patients		Control
		*N* = 39		*N* = 25
Age (Mean ± SD)		57 ± 9.8 years		52.3 ± 10.9 years
Recurrence incidence		*N*	%	
	Yes	15	38.5	
	No	24	61.5	
Death incidence		*N*	%	
	Yes	10	25.6	
	No	29	74.4	
Smoking		*N*	%	
	Yes	23	59	
	No	16	41	
Grades	Grade 1	6	15.38%	
	Grade 2	14	35.89%	
	Grade 3	19	48.71%	
Immunophenotyping	HR− /HER2+	2	5.1%	
	HR+ /HER2−	15	38.5%	
	Triple positive	15	38.5%	
	Triple negative	7	17.9%	

The receptor status of breast cancer plays a crucial role in determining prognosis and treatment options. Hormone receptor‐positive (HR+ /HER2− ) tumors, which constituted 38.5% of our patient group, are generally associated with a better prognosis and respond well to endocrine therapy such as tamoxifen or aromatase inhibitors [29]. Triple‐positive tumors (HR+ /HER2+ ), also representing 38.5%, may benefit from a combination of hormonal therapy and anti‐HER2 targeted therapies like trastuzumab.

On the other hand, triple‐negative breast cancer (TNBC), which accounted for 17.9% of cases, is considered more aggressive, with limited targeted treatment options and a higher risk of recurrence. These patients often require chemotherapy as the mainstay of treatment. Finally, the HER2‐positive but hormone receptor‐negative subtype (5.1%) is also aggressive but responds well to HER2‐targeted therapies.

Our findings align with existing literature, where hormone receptor‐positive cases are the most prevalent, while triple‐negative cases, although less frequent, pose significant therapeutic challenges. The distribution of receptor subtypes in our cohort is consistent with global breast cancer patterns and highlights the need for personalized treatment strategies based on receptor status.

### 3.2 Comparison Between the Patient Group and the Control Group

**Figure 1:**
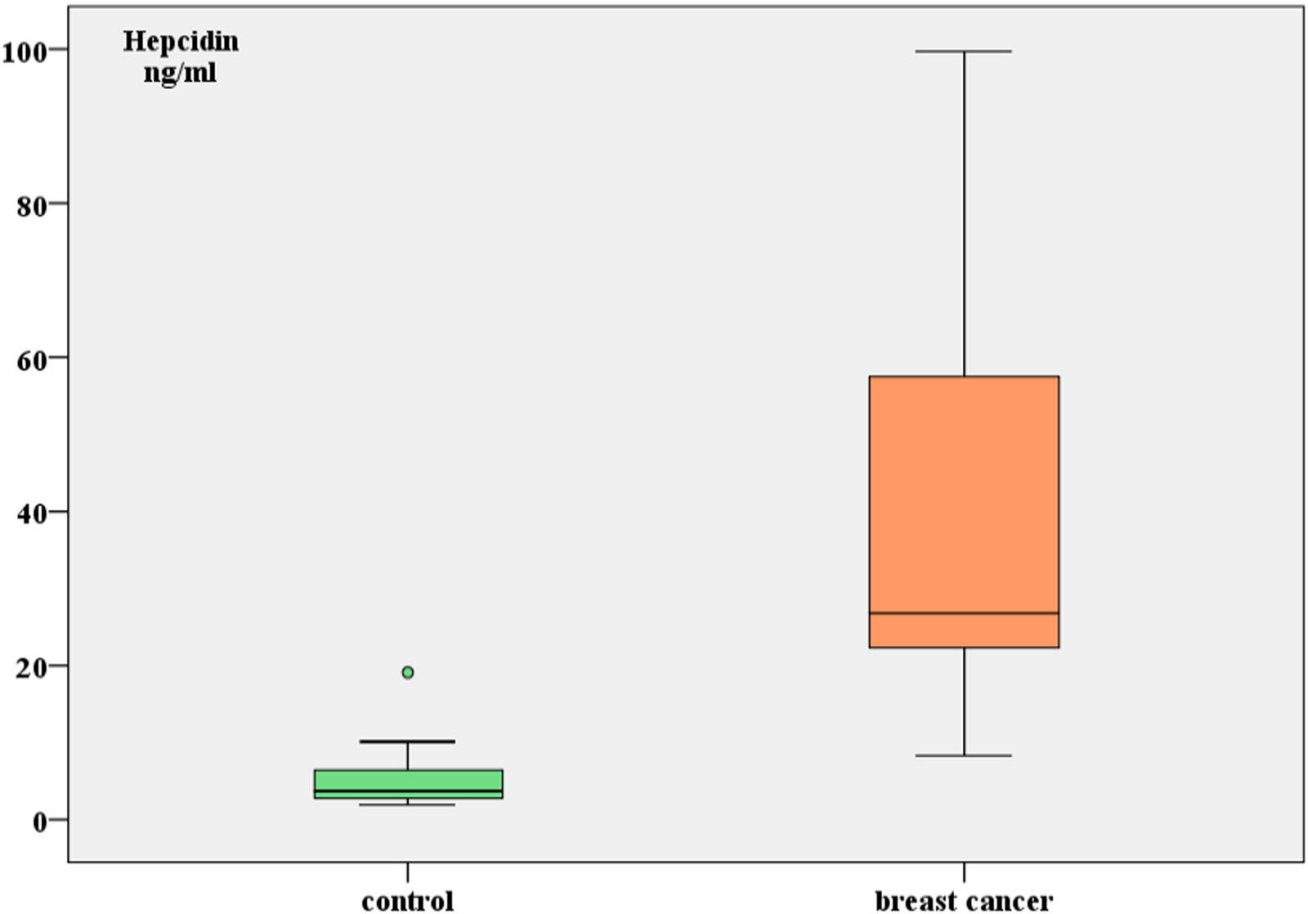
Comparison of hepcidin between patients and healthy control.

Serum CRP, Fe and hepcidin were tested for all participants. Hb, WBC, RBC and MCV were also considered. As shown in (Table 2 and Figure 1) CRP and hepcidin levels were significantly elevated in the patient group compared to controls (39.05 ± 22.9 vs. 2.63 ± 1.57 mg/L, *p* < 0.05) and (39.36 ± 28.12 vs. 5.56 ± 4.87 ng/mL, *p* < 0.05), respectively. In contrast, Fe and Hb levels were significantly reduced in breast cancer patients compared to controls (80.89 ± 35.8 vs. 95.07 ± 24.02, *p* < 0.05) and (11.63 ± 1.33 vs. 13.74 ± 0.97, *p* < 0.05). No significant difference was found between the two groups in term of WBC, RBC or MCV.

**Table 2:** Comparison of hematological and biochemical parameters between patients and healthy control.

Mean ± SD							
	Hepcidin (ng/mL) Mean ± SD	CRP (mg/L) Mean ± SD	Fe (ug/dL) Mean ± SD	Hb (g/dL) Mean ± SD	WBC (*103/UL) Mean ± SD	RBC (*10 6/UL) Mean ± SD	MCV (FL) Mean ± SD
Patients	39.36 ± 28.12	39.05 ± 22.9	80.89 ± 35.8	11.63 ± 1.33	4.17 ± 1.26	4.18 ± 0.56	111.58 ± 30.3
Healthy controls	5.56 ± 4.87	2.63 ± 1.57	95.07 ± 24.02	13.74 ± 0.97	5.54 ± 3.15	4.475 ± 0.38	80.14 ± 7.46
*p* value	**0.0000006**	**0.0034**	**0.00009**	**0.00002**	0.20	0.0893	**0.09**

*Note:* Bold values indicate statistical significance at *p* < 0.05.

WBC, RBC, and MCV levels provide important insights into the hematological status of breast cancer patients. While no significant difference was found in WBC counts between groups, RBC levels were lower in breast cancer patients, possibly due to cancer‐related anemia. Cancer‐induced inflammation and iron metabolism dysregulation, driven by elevated hepcidin, may contribute to reduced RBC production [30]. Moreover, while MCV levels did not show significant changes between patients and controls, this parameter may be affected by underlying iron metabolism alterations, chronic inflammation, or the early effects of disease progression.

### 3.3 Comparison Between Recurrenced and Nonrecurrenced Breast Cancer Patients Groups

**Figure 2:**
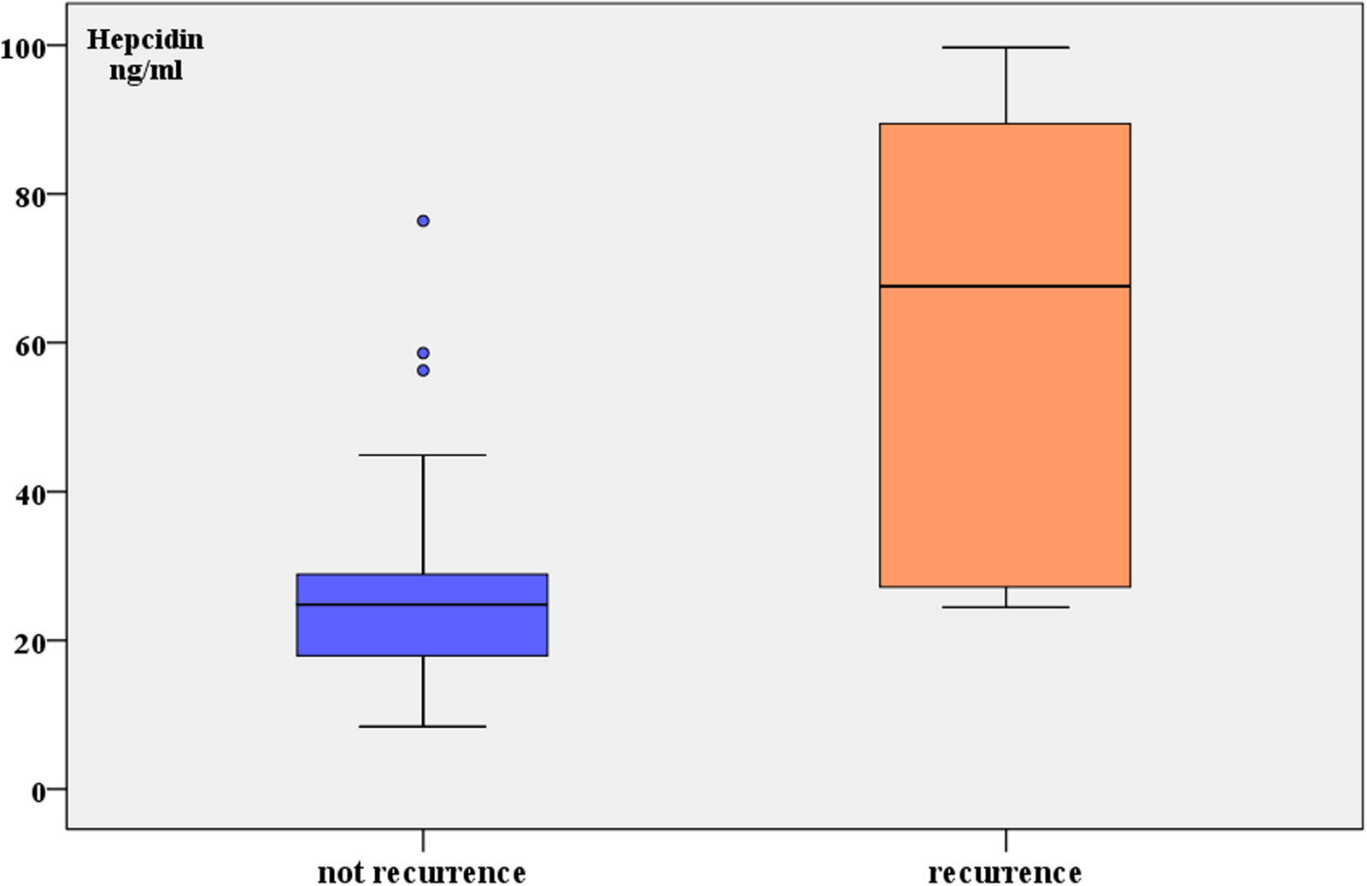
Comparison of serum hepcidin levels between the recurrence and nonrecurrence groups.

**Figure 3:**
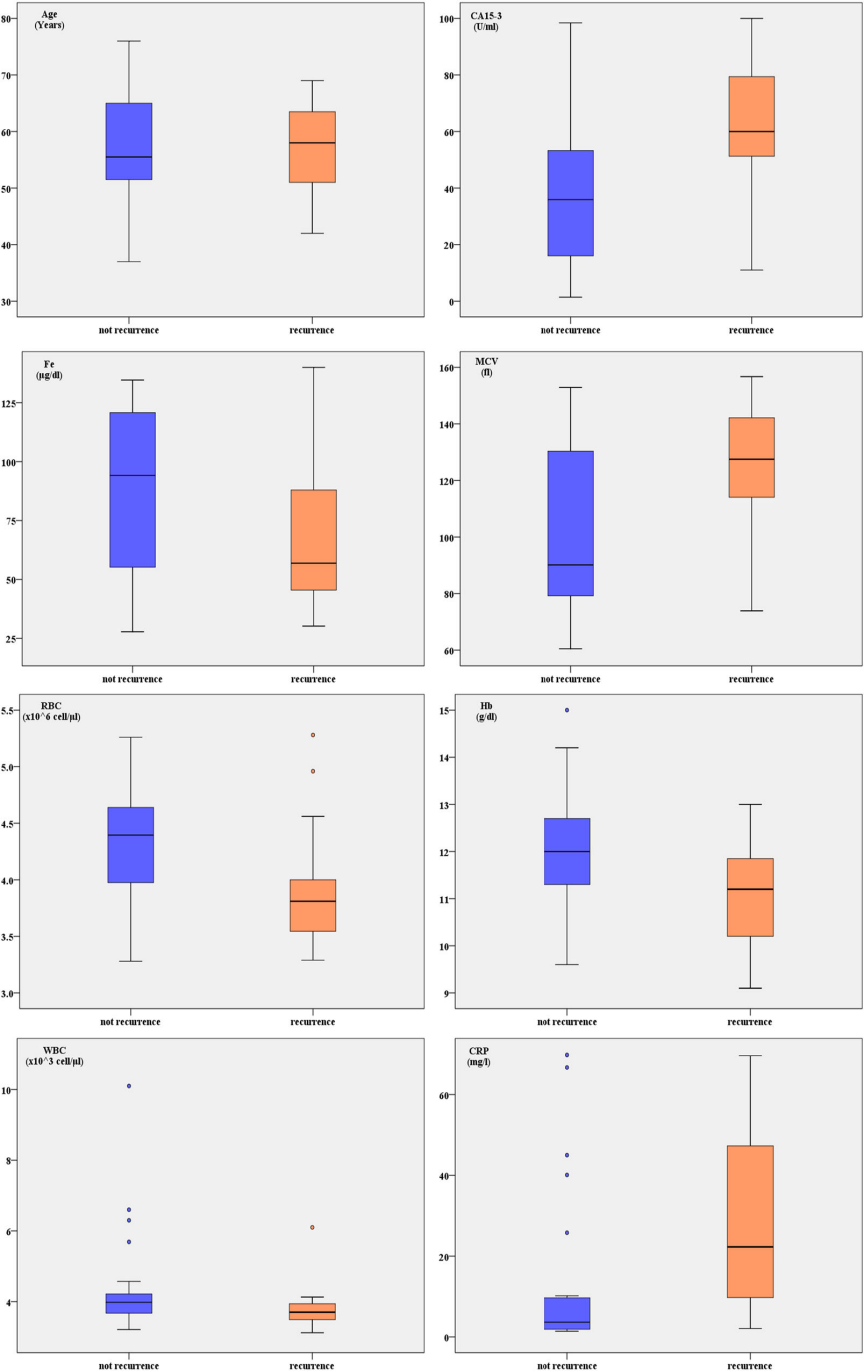
Comparison of age, CA 15‐3, CRP, WBC, RBC, Fe, Hb and MCV between recurrenced and nonrecurrenced breast patients.

CA15‐3 levels, CRP, and MCV were significantly higher in the recurrenced group compared to the nonrecurrenced (60.9 ± 30.6 vs. 39.8 ± 30.6, *p* = 0.033), (29.2 ± 24 vs. 13.5 ± 20.5, *p* = 0.009) and (125.5 ± 23.5 vs. 102.9 ± 31.2, *p* = 0.028), respectively, while Hb and RBC were significantly lower in the recurrenced group compared to the non‐recurrenced (11 ± 1.2 vs. 12 ± 1.3, *p* = 0.026) and (3.9 ± 0.6 vs. 4.3 ± 0.5, *p* = 0.040). The recurrenced group showed increased serum hepcidin compared to the non‐recurrenced (58.79 ± 16.51 vs. 27.22 ± 32.27 ng/mL, *p* = 0.0009). There was no statistically significant difference between the two groups in terms of age, Fe or WBC (Table 3) (Figures 2 and 3).

**Table 3:** Comparison of study parameters between (not recurrence) and (recurrence) groups.

	Median (SD)		*p* value
Progression (*N*)	Not recurrence (24)	Recurrence (15)	
Age (Years)	55.5 (10.8)	58 (8.4)	0.98
Hepcidin (ng/mL)	24.75 (16.5)	67.5 (32.3)	**0.0009**
CA15‐3 (U/mL)	35.9 (30.6)	60 (26.1)	**0.033**
RBC (×10^6^ cell/μL)	4.395 (0.5)	3.81 (0.6)	**0.04**
Hb (g/dL)	12 (1.3)	11.2 (1.2)	**0.026**
MCV (fl)	90.15 (31.2)	127.5 (23.5)	**0.028**
CRP (mg/L)	3.65 (20.5)	22.3 (23.9)	**0.009**
Fe (μg/dL)	94.15 (35.7)	56.9 (34.4)	0.15
WBC (×10^3^ cell/μL)	3.98 (1.5)	3.7 (0.7)	0.059

*Note:* Bold values indicate statistical significance at *p* < 0.05.

The decrease in RBC and Hb levels in the recurrenced group suggests a stronger impact of cancer progression on anemia development. Elevated hepcidin levels in these patients may play a role in iron sequestration, leading to impaired erythropoiesis and a worsening anemic state [30]. Additionally, the significant increase in MCV in the recurrenced group might indicate a compensatory response to anemia or altered erythropoiesis due to chronic disease and systemic inflammation. These findings suggest that RBC and MCV changes could serve as potential indicators of disease recurrence in breast cancer patients.

### 3.4 Study Variables and Hepcidin Serum Levels According to Breast Cancer Stage

Table 4 presents data across three stages, with each stage having 13 observations. Key findings include:

- **Hepcidin:** Increases significantly from Stage I to III (*p* = 0.0000002).
- **Age:** Median age rises across stages (*p* = 0.000011).
- **RBC Count:** Decreases progressively (*p* = 0.000059).
- **Hemoglobin:** Decreases significantly with stage progression (*p* = 0.000006).
- **CA15‐3:** Levels rise significantly from Stage I to III (*p* = 0.000000).
- **CRP:** Increases markedly across stages (*p* = 0.000012).
- **Iron:** Decreases significantly (*p* = 0.000001).
- **WBC Count:** Shows a significant decrease (*p* = 0.000106).
- **MCV:** Increases significantly with disease stage (*p* = 0.000015).

**Table 4:** Differences in study parameters between tumor stages.

	Median (SD)			*p* value
Stage	I	II	III	
(*N*)	13	13	13	
Hepcidin (ng/mL)	18.7 (6.1)	26.4 (1.9)	76.4 (22)	**0.0000002**
Age (Years)	51 (7.5)	59 (6.7)	64 (7.8)	**0.000011**
RBC (×10^6^ cell/μL)	4.6 (0.4)	3.98 (0.4)	3.58 (0.4)	**0.000059**
Hb (g/dL)	12.8 (1.3)	11.8 (0.8)	10.3 (0.7)	**0.000006**
CA15‐3 (U/mL)	14.2 (10.2)	46.9 (8.3)	89.4 (16)	**0.000000**
CRP (mg/L)	2.5 (1)	8.9 (9.7)	45 (21.5)	**0.000012**
Fe (μg/dL)	123.6 (11.5)	81.2 (23.9)	45.2 (13.7)	**0.000001**
WBC (×10^3^ cell/μL)	4.02 (0.6)	3.94 (1.9)	3.34 (0.3)	**0.000106**
MCV (fl)	83.9 (19.4)	118.4 (22.3)	146.3 (19.4)	**0.000015**

*Note:* Bold values indicate statistical significance at *p* < 0.05.

**Figure 4:**
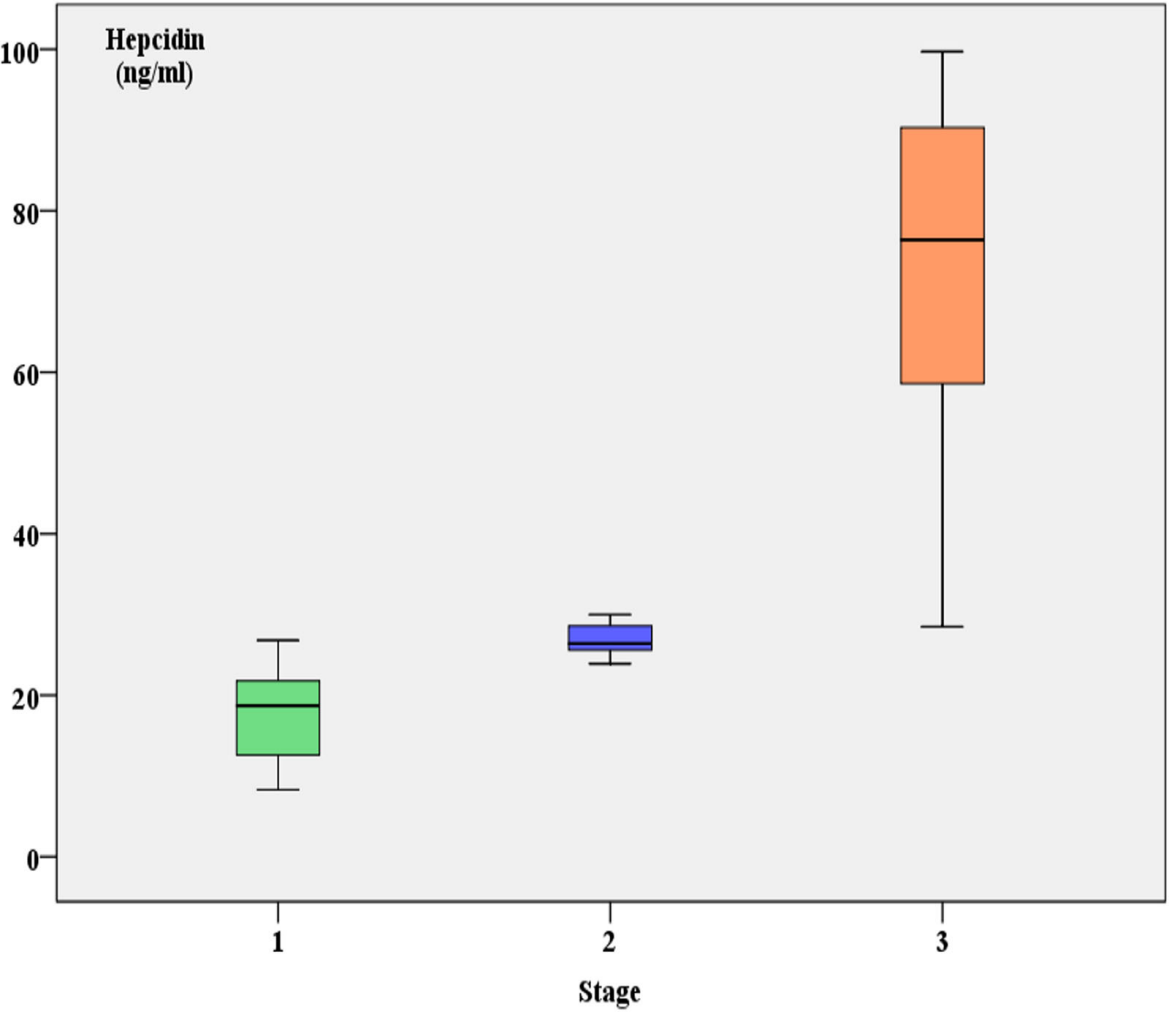
Comparison of hepcidin levels between breast cancer stages.

**Figure 5:**
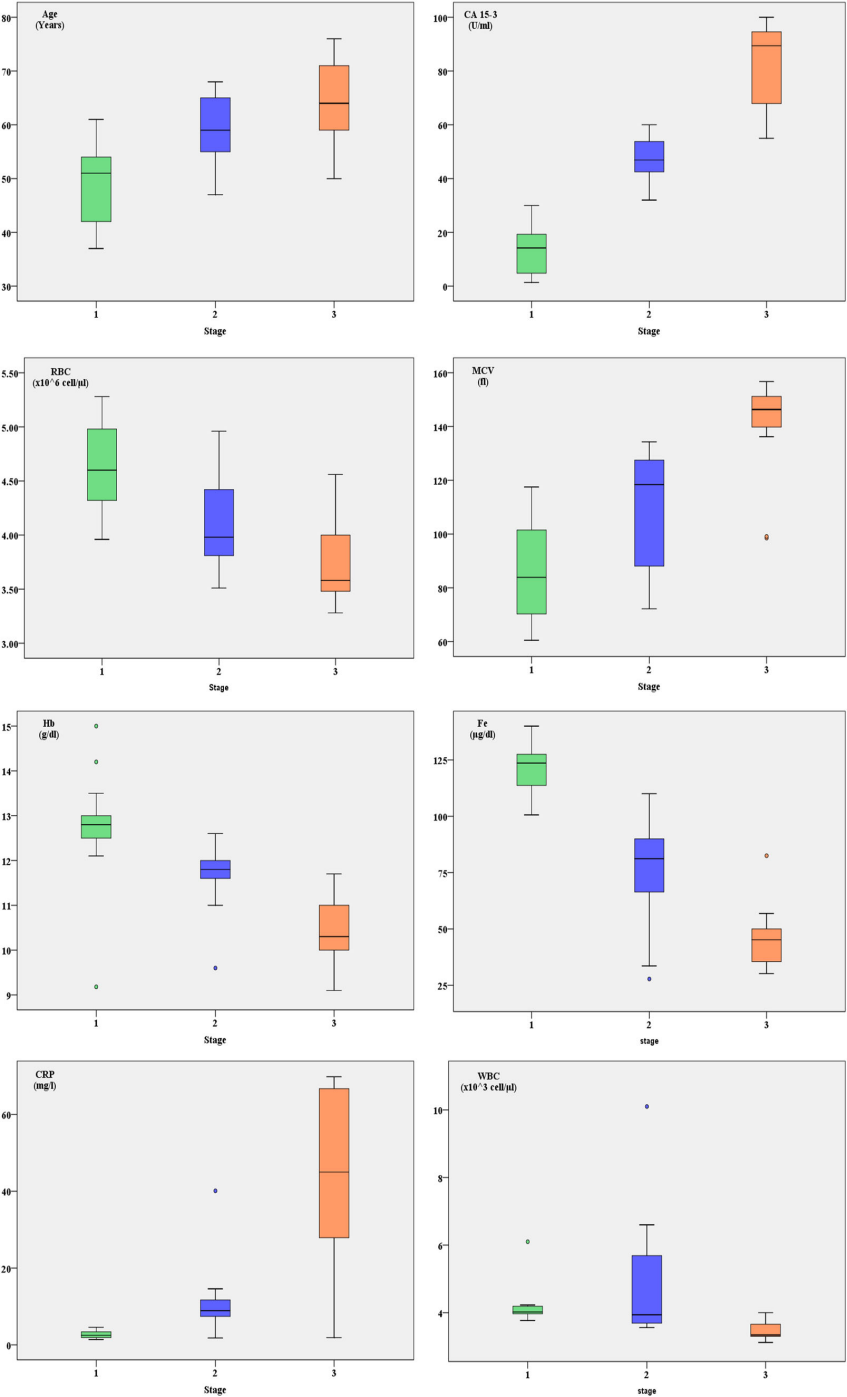
Comparison of study variables across the three stages of breast cancer.

These results indicate significant changes in these parameters as the disease advances through the stages (Figures 4 and 5).

The progressive decline in RBC count and Hb levels across tumor stages supports the hypothesis that advanced breast cancer is associated with worsening anemia. This is likely due to increased inflammatory cytokine production, altered iron homeostasis, and enhanced hepcidin‐mediated iron sequestration. The observed increase in MCV with disease progression could be attributed to compensatory mechanisms in response to hypoxia or chronic anemia. These findings further highlight the impact of systemic inflammation and iron metabolism dysregulation on hematological parameters in breast cancer patients [31].

### 3.5 The Diagnostic and Prognostic Significance of Serum Hepcidin in Breast Cancer Patients

**Figure 6:**
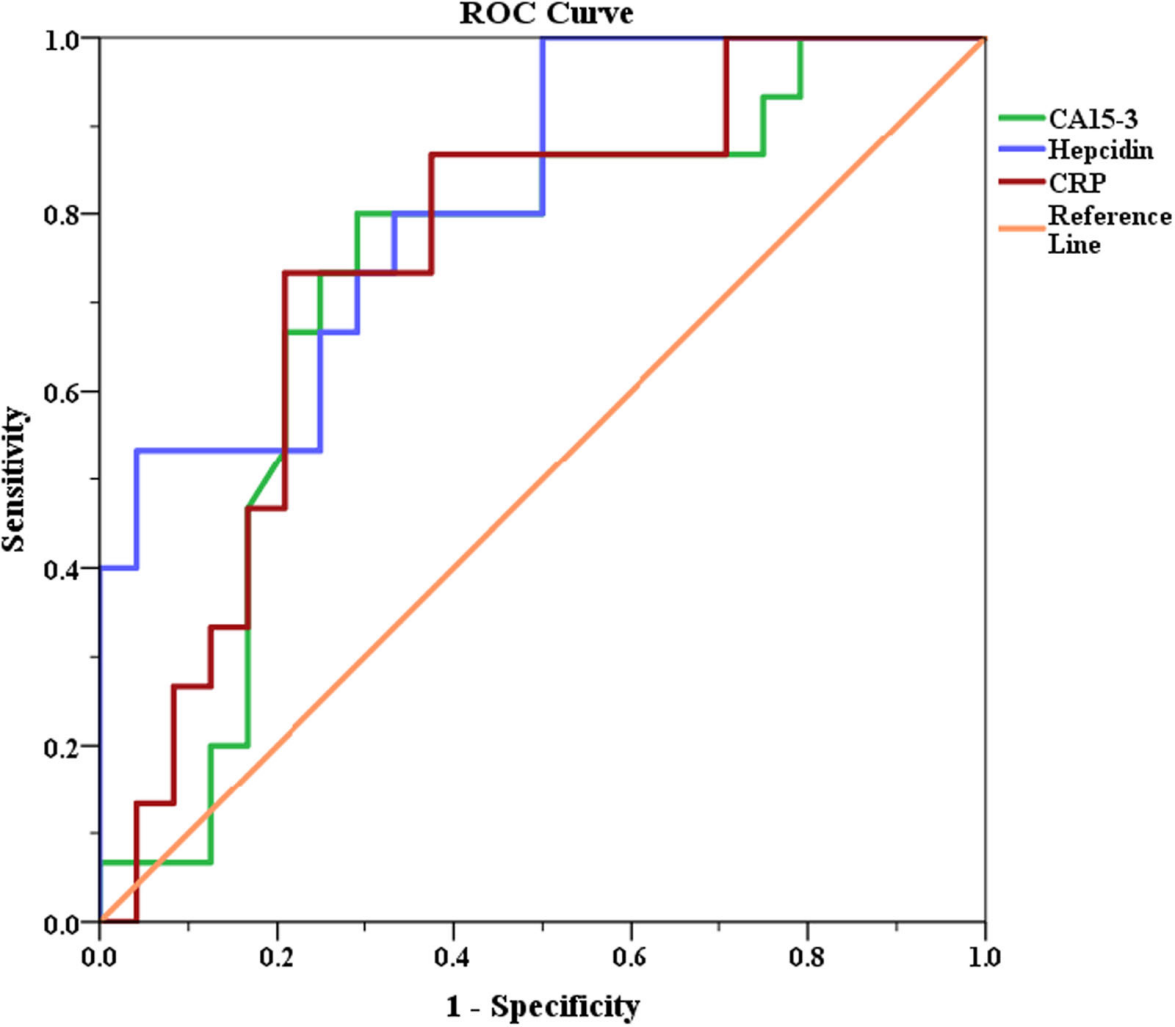
The receiver–operating characteristic (ROC) curve of hepcidin, CA 15‐3, and CRP for predicting recurrence in breast cancer patients.

The AUC for CRP is 0.753 with a cut‐off of 10.95 mg/L, showing a sensitivity of 73.3% and a specificity of 79.2% (*p* = 0.009). The AUC for CA15‐3 is 0.726 with a cut‐off of 47.8 U/mL, demonstrating a sensitivity of 80% and a specificity of 70.8% (*p* = 0.019). ROC curve analysis demonstrated that hepcidin had a strong discriminatory ability for breast cancer recurrence (AUC = 0.819, 95% CI: 0.75–0.89, *p* < 0.001). A cut‐off value of 26.6 ng/mL yielded a sensitivity of 80% and a specificity of 66.7% for predicting recurrence (Figure 6). Based on this value, breast cancer patients were divided into two groups according to hepcidin levels; Group 1: Low hepcidin (Hepcidin ≤ 26.6 ng/mL) comprising 19 patients, and Group 2: High hepcidin (Hepcidin > 26.6 ng/mL) comprising 20 patients (Table 5).

**Table 5:** Sensitivity, specificity, cutoff point, and P value for each of hepcidin, CRP, and CA15‐3.

	*p* value	Cut‐off	AUC	Sensitivity	Specificity
Hepcidin ng/mL	0.001	26.6	0.819	80%	66.7%
CA 15‐3 U/mL	0.019	47.8	0.726	80%	70.5%
CRP mg/L	0.009	10.95	0.753	73.3%	79.2%

**Figure 7:**
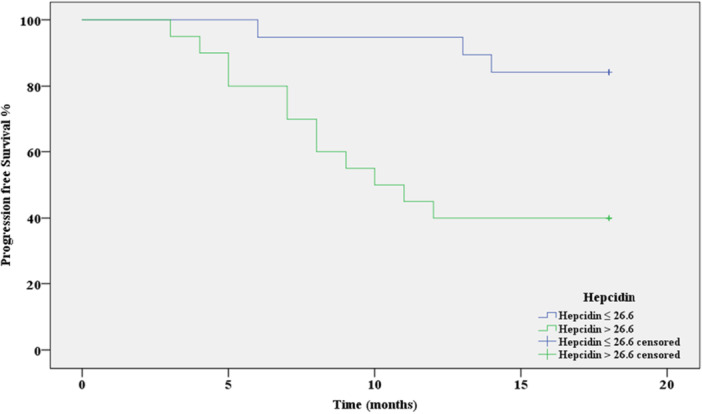
The Kaplan‐Meier test (PFS), cut‐off = 26.6, *p* = 0.0027, AUC = 0.819.

The Kaplan‐Meier test was performed to investigate the importance of serum hepcidin in predicting recurrence in breast cancer patients. Among the 20 patients of Group 2 (Hepcidin > 26.6 ng/mL), 12 patients suffered disease recurrence, while in Group 1 (Hepcidin ≤ 26.6 ng/mL), only 3 out of 19 patients recurrenced during the follow‐up period. The progression free survival (PFS) mean and rate (16.9 months and 84%, respectively) in Group 1 were higher than the PFS mean and rate in Group 2 (11.6 months and 40%, respectively), and this difference was statistically significant (*p* = 0.0027) (Figure 7) (Table 6).

**Table 6:** Patients progression free survival (PFS) mean and rate according to hepcidin levels.

	Number of recurrences	Number of PFS cases	PFS rate	PFS mean (standard error)	Confidence interval for the mean			*p* value
					95% CI		Log rank Chi‐Square	
					Maximum	Minimum		
**Group 1:**	3	16	84%	16.9 (0.669)	18.2	15.6	8.968	**0.0027**
**Hepcidin** ≤ **26.6 ng/mL**								
***n*** = **19**								
**Group 2:**	12	8	40%	11.6 (1.250)	14.1	9.2		
**Hepcidin > 26.6 ng/mL**								
***n*** = **20**								

*Note:* Bold value indicates statistical significance at *p* < 0.05.

**Figure 8:**
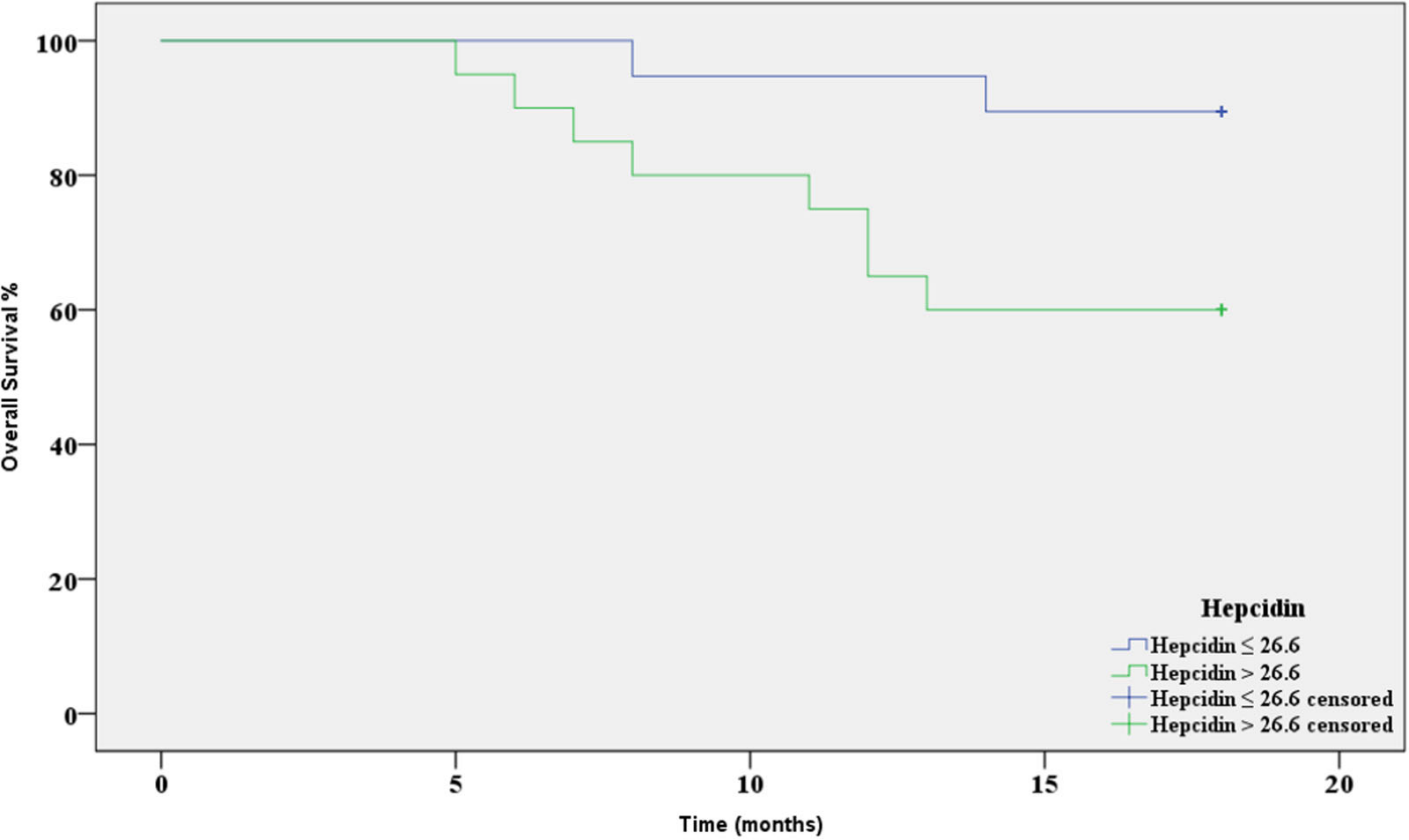
The Kaplan‐Meier test (OS), cut‐off = 26.6, *p* = 0.032, AUC = 0.819.

The Kaplan‐Meier test was also performed to estimate the overall survival (OS) rate in the two hepcidin groups (Figure 8). Only two out of 19 patients with low hepcidin (Group 1) died, while 8 out of 20 patients died in the high hepcidin group (Group 2).

The higher mortality rate in patients with elevated hepcidin levels (40%) compared to those with lower levels (10.5%) suggests a potential association between hepcidin overexpression and poorer survival outcomes. Although specific causes of death were not systematically recorded, previous studies have linked elevated hepcidin levels with increased systemic inflammation, anemia of chronic disease, and iron dysregulation—all of which may contribute to cancer progression and worsened clinical outcomes. Furthermore, high hepcidin levels might reflect more aggressive tumor biology or a higher inflammatory burden, both of which have been associated with decreased survival in breast cancer patients.

The OS mean and rate in Group 1 (17.26 months and 89.5%, respectively) were higher than those of Group 2 (14.5 months and 60%, respectively), and the difference was statistically significant (*p* = 0.032) (Table 7).

**Table 7:** Patients overall survival (OS) mean and rate according to hepcidin levels.

	The number of deaths	Number of (OS) cases	(OS) rate	OS Mean (standard error)	Confidence interval for the mean		Log rank Chi‐Square	*p* value
					95% CI			
					Maximum	Minimum		
**Group 1:**	2	17	89.50%	17.26 (0.54)	18.3	16.2	4.6	**0.032**
**Hepcidin** ≤ **26.6 ng/mL**								
***n*** = **19**								
**Group 2:**	8	12	60%	(1) 14.5	16.5	12.4		
**Hepcidin > 26.6 ng/mL**								
***n*** = **20**								

*Note:* Bold value indicates statistical significance at *p* < 0.05.

## 4 Discussion

Breast cancer ranks among the most prevalent malignancies worldwide [1]. Hepcidin is a hormone produced in the liver and is considered the main regulator of iron levels in the body [11]. Studies have shown that hepcidin levels are elevated in many benign diseases and cancers, including breast cancer [32, 33]. However, the mechanism by which serum hepcidin rises in breast cancer patients still lacks evidence.

### 4.1 Hepcidin in Breast Cancer: Impact on Iron Homeostasis and Therapeutic Implications

The results of this study showed higher levels of hepcidin in breast cancer women (*p* ≤ 0.05) than in healthy controls. These findings are consistent with previous studies reporting an elevated serum hepcidin in cancers [26, 34, 35]. In fact, hepcidin is suggested to play multiple roles in cancer biology through various proposed mechanisms; Hepcidin modulates iron homeostasis by binding to the iron export protein ferroportin, causing its degradation. This reduces the release of iron from macrophages and enterocytes into the bloodstream, leading to cellular iron retention [36]. In cancer cells, elevated hepcidin levels results in increased intracellular iron retention, promoting tumor growth and proliferation by supporting the high metabolic demands of rapidly dividing cells [35]. This mechanism is critical for the survival and progression of cancers, including breast cancer, as hepcidin‐induced iron accumulation enhances the activity of iron‐dependent enzymes and proteins involved in the synthesis and repair of DNA, mitochondrial respiration, and cellular proliferation [34, 35]. Additionally, iron contributes to oxidative stress within cells, which can lead to genetic mutations and tumor heterogeneity, further driving cancer progression and metastasis [37]. Hepcidin affects not only cancer cells but also the tumor microenvironment [38, 39]. By regulating iron availability, hepcidin can influence immune cell function, angiogenesis, and the inflammatory response [38, 39, 40]. Consistent with this, iron‐loaded macrophages can exhibit a protumorigenic phenotype, supporting tumor growth and immune evasion [38, 39, 41]. The role of hepcidin in modulating iron levels in the tumor microenvironment is therefore crucial to create a favorable niche for cancer development [42]. Due to the critical role of hepcidin‐induced iron overload in cancer cell development, hepcidin levels have been suggested as a predictive biomarker of cancer response to treatment. For example, iron chelation therapy, which reduces iron availability, has shown potential in enhancing the effectiveness of anticancer drugs by depriving cancer cells of the iron necessary for their growth [43, 44]. Conversely, treatments that inadvertently increase hepcidin levels could potentially diminish therapeutic outcomes by promoting iron retention and tumor progression [34, 43, 44]. These findings underscore the pivotal role of hepcidin in breast cancer progression through its regulation of iron metabolism and tumor microenvironment dynamics. Understanding hepcidin's influence could pave the way for novel biomarkers and therapeutic strategies tailored to improve treatment outcomes in breast cancer patients.

### 4.2 Hepcidin Levels in Breast Cancer Recurrence: Implications for Iron Regulation and Prognosis

Comparing hepcidin levels between the two sub‐groups of recurrenced and nonrecurrenced patients, it was found that the hepcidin levels were higher and statistically significant in the recurrenced group compared to the non‐recurrenced. Recurrence in breast cancer usually occurs when the cancer comes back after initial treatment, either in the same location or in other parts of the body. The elevated hepcidin levels in recurrenced patients may be a results of several cancer related factors/mechanisms. Firstly, the tumor microenvironment is known to induce a chronic inflammatory state, characterized by increased production of cytokines such as interleukin‐6 (IL‐6) and other acute‐phase inflammatory proteins such as CRP [45]. Hepcidin synthesis from liver cells is upregulated in response to inflammatory signals, leading to intracellular iron sequestration and subsequent hypoferremia [46, 47]. In this study, the sustained inflammatory response within the tumor microenvironment of recurrenced patients may have contributed to the observed elevation of hepcidin levels. Consistent with this hypothesis, CRP levels in this study were statistically increased in the recurrenced group compared to the non‐recurrenced, suggesting that CRP could potentially be a positive regulator of hepcidin in breast cancer patients. Furthermore, serum irons levels were reduced in the recurrenced group compared to the non‐recurrenced, although not significant. Additionally, cancer‐related anemia, a common complication in cancer patients, could also be affected and/or could itself influence hepcidin regulation. Tumors often sequester iron for their own proliferation, leading to decreased iron availability for erythropoiesis and subsequent anemia. Elevated hepcidin levels in recurrenced patients may reflect an attempt by the body to sequester iron away from the tumor, thereby worsening the anemic state of these individuals [46]. In this study, hemoglobin concentration was significantly lower in recurrenced than in non‐recurrenced patients, which correlated with the elevated serum hepcidin levels observed in recurrenced patients.

Moreover, the lingering effects of chemotherapy may contribute to the dysregulation of iron metabolism observed in recurrenced patients. Chemotherapeutic agents can induce inflammation and oxidative stress, further stimulating hepcidin synthesis. Thus, the higher hepcidin levels observed in recurrenced patients could, in part, be attributed to the cumulative effects of chemotherapy [46, 47]. The findings of this study are consistent with previous studies that have reported elevated hepcidin levels in cancer patients, particularly those experiencing disease recurrence or progression [48, 49, 50]. These studies have emphasized the complex interplay between iron metabolism, inflammation, and cancer progression, highlighting the potential utility of hepcidin as a biomarker for disease monitoring and prognosis. In contrast to this study results results, some studies have reported conflicting results regarding hepcidin levels in recurrenced versus non‐recurrenced cancer patients [51, 52]. These discrepancies may stem from differences in patient populations, cancer types, treatment regimens, or assay methodologies. Further research is needed to reconcile these inconsistencies and establish the robustness of hepcidin as a cancer prognostic marker.

### 4.3 Biomarker Differences Between Recurrenced and Nonrecurrenced Breast Cancer: Insights and Implications

This study observed significant differences in several biomarkers between the recurrenced and nonrecurrenced groups. Specifically, CA15‐3, CRP, and MCV were higher in the recurrenced group, while Hb levels and RBC counts were lower in the recurrenced group. No significant differences were found in age, serum iron levels, and WBC counts between the two groups. These findings provide valuable insights into the biological changes associated with breast cancer recurrence and align with or contrast existing literature in various ways.

CA15‐3 is a well‐established tumor marker used in monitoring breast cancer progression. Elevated CA15‐3 levels in patients with recurrenced breast cancer indicate that the cancer has returned after initial treatment, either locally or elsewhere in the body [53]. Higher CA15‐3 levels reflect greater tumor burden and more advanced disease stage, which are often associated with increased risk of disease recurrence or disease progression [53]. Therefore, this biomarker can be useful for monitoring disease progression and predicting the risk of disease recurrence in breast cancer patients.

In this study, This study demonstrated elevated CA15‐3 levels in the recurrenced group, and this is consistent with previous studies reporting its association with disease recurrence and metastasis. In this sense, Duffy et al. gave evidence of the utility of CA15‐3 as a marker for detecting recurrence in breast cancer patients [54]. Similarly, a study by Uehara et al. demonstrated that higher CA15‐3 levels correlated with poorer prognosis and recurrence in breast cancer patients [55], reinforcing thus the observations of this study.

CRP is a general inflammatory marker that can be elevated in various conditions, including cancer [56]. Mean CRP was within normal range in healthy controls (2.63 g/dL), while in breast cancer patients, high CRP levels (39.05 g/dL) may refer to a significant association of the inflammatory process with disease progression [57]. In recurrenced breast cancer, CRP levels were significantly higher than levels in non‐recurrenced patients due to more aggressive disease with higher tumor burden and poorer prognosis. This result is supported by the work of Pierce et al. which discussed the role of chronic inflammation in promoting tumor growth and metastasis [58].

Additionally, the increased MCV observed in this study may reflect compensatory mechanisms in response to hypoxia or anemia, common in advanced cancer stages [59]. However, contrary findings were reported by previous studies [60, 61, 62] where no significant changes in MCV were noted between recurrenced and non‐recurrenced patients, suggesting potential variability in patient response or difference in study design and population. These findings highlight the clinical relevance of biomarkers like CA15‐3 and CRP in predicting breast cancer recurrence and monitoring disease progression. Understanding these biological changes could aid in refining treatment strategies and improving outcomes for breast cancer patients.

### 4.4 Iron Metabolism and Anemia in Breast Cancer: Insights Into Disease Progression

The decrease in serum iron and Hb in breast cancer patients compared to controls may signify anemia induced by the cancer itself, since the patients had not received any treatment at the time of blood sampling. Tumors can release inflammatory cytokines that affect bone marrow function, impairing the production of new red blood cells [63, 64]. In addition, certain breast cancer subtypes are associated with increased angiogenesis (formation of new blood vessels), which may also contribute to anemia by competing for oxygen transport in the tumor microenvironment [65, 66, 67]. Moreover, Hb and RBCs were significantly decreased in recurrenced compared to non‐recurrenced patients, meaning that anemia could be a predictor of poor prognosis in patients with breast cancer. These results are consistent with the findings of Ludwig et al. who reported that anemia is prevalent in recurrenced cancer patients due to multiple factors, including bone marrow suppression and chronic disease [68]. However, a study by Groopman and Itri demonstrated that although anemia is common during active treatment, its persistence upon recurrence varies, emphasizing the need for individualized patient management [69].

Anemia in breast cancer patients may be attributed to increased hepcidin levels, which lead to iron dysregulation and are associated with disease development and progression. Hepcidin, a peptide hormone regulating iron homeostasis, is often elevated in response to inflammation and cancer, driven by cytokines such as interleukin‐6 (IL‐6) [62, 70]. Elevated hepcidin reduces iron absorption from the intestine and traps iron in macrophages, resulting in reduced serum iron levels and contributing to anemia of chronic disease (ACD) or anemia of inflammation (AI) commonly observed in cancer patients [70]. Studies have shown that IL‐6, frequently increased in cancer, induces hepcidin production, leading to hypoferremia and anemia [70]. This mechanism highlights the intricate link between cancer‐related inflammation, hepcidin regulation, and anemia, underscoring the impact of systemic inflammation on iron metabolism in breast cancer patients [62, 70].

The lack of significant differences in age, serum iron levels, and white blood cell counts in relation to patients' susceptibility to recurrence indicates that these factors may not have a direct impact on recurrence status in breast cancer. This is consistent with the findings of Chou et al. who reported no significant age‐related differences in recurrence rates [71]. Similarly, studies have shown no direct correlation between serum iron or WBC and breast cancer recurrence, suggesting that other biomarkers might be more relevant for monitoring disease progression [62, 72, 73, 74].

### 4.5 Iron Regulation and Biomarker Changes in Breast Cancer Progression

This study demonstrated that Elevated hepcidin levels in breast cancer patients were associated with disease progression, suggesting its potential role as a biomarker. Specifically, hepcidin levels were significantly higher in stage III breast cancer patients than patients in stage I and II, and likewise, levels in stage II patients were significantly higher than in stage I patients.

In the advanced stages of cancer, the tumor requires more nutrients and minerals, including iron. To sustain the rapid proliferation of cancer cells, this may lead to the stimulation of the inflammatory response and an increase in hepcidin levels, aiming to modify iron distribution to meet the tumor's demands [75].

In the advanced stages of cancer, hepcidin levels often increase due to both the tumor itself and the body's inflammatory response [76]. Hepcidin, regulates iron homeostasis by inhibiting iron absorption from the intestine and iron release from macrophages [77]. In cancer, especially advanced stages, inflammatory cytokines like interleukin‐6 (IL‐6) are elevated, which in turn stimulates hepcidin production [77]. Tumors themselves can also secrete hepcidin or induce its production indirectly through inflammatory pathways [76]. As the tumor progresses, the inflammatory response intensifies, further increasing hepcidin levels and contributing to anemia commonly observed in cancer patients [77]. This anemia is characterized by iron sequestration in macrophages and reduced iron availability for erythropoiesis, leading to a state of functional iron deficiency [76]. Several previous studies reported similar results; George et al. (2021) have shown that hepcidin levels were significantly elevated in patients with more advanced breast cancer stages [78]. A study by Pan et al. also found that hepcidin levels were higher in breast cancer patients with stage II and III than in those with stage I [30]. However, contradictory results were reported by Ma et al. who claimed no significant difference in hepcidin levels between different stages of breast cancer, suggesting that the correlation between hepcidin levels and cancer stage may not be straightforward, and could be influenced by other factors such as patient iron status, inflammation, and comorbidities [79].

The disparity between studies may result from differences in the demographic characteristics of the study sample, and the presence of confounding factors such as inflammation and iron metabolism disorders. Future research should focus on larger, more homogenous cohorts and consider these confounding variables to better elucidate the relationship between hepcidin levels and breast cancer progression. In our study, we examined various hematological and biochemical markers across different stages of breast cancer. Our findings revealed significant alterations in the levels of CRP, CA 15‐3, MCV, Hb, RBC and Fe as the disease progressed from stage I to stage III.

Elevated CRP levels indicate systemic inflammation, which is commonly associated with advanced stages of cancer due to the inflammatory response elicited by the tumor and the surrounding tissue [80]. CA15‐3, as mentioned previously, is a well‐known tumor marker for breast cancer, with higher levels correlating with tumor burden and progression [81]. An increase in MCV could reflect changes in erythropoiesis, possibly due to chemotherapy or the body's response to chronic disease [82].

Anemia, characterized by a reduction in hemoglobin and RBC counts, is common in cancer patients, especially in advanced stages, and can result from a variety of factors including chronic illness, nutritional deficiencies, and myelosuppressive therapies [68]. Lower serum iron levels in late stages may further be attributed to the body's attempt to limit iron availability to cancer cells through mechanisms involving hepcidin secretion and iron sequestration [83].

Interestingly, while WBC count was significantly lower in stage III compared to stages II and I, there was no significant difference in WBC count between stages I and II. This reduction in leukocytes count in the later stages could be due to bone marrow suppression from the cancer itself or as a side effect of treatments like chemotherapy [84].

These results are almost consistent with previous research. A study by Al‐Azawi et al. found elevated CRP and CA15‐3 levels in advanced breast cancer, supporting this study results [85]. Similarly, research by Knight et al. (2014) reported anemia and low serum iron in later stages of breast cancer, consistent with this study observations [86]. However, Sharma et al. did not find significant changes in WBC counts at different cancer stages, suggesting that this parameter might be influenced by additional factors such as infection or specific treatment regimens [87].

Taken together, this study indicates that CRP, CA15‐3, and MCV levels increase, while hemoglobin, RBC count, and serum iron decrease with advancement of breast cancer. These findings highlight the complex interplay between cancer progression and systemic physiological responses, and give insight into the potential utility of these markers in monitoring disease progression and tailoring patient management strategies.

### 4.6 Serum Hepcidin Levels as Prognostic Markers in Breast Cancer

This study put emphasis on the prognostic significance of serum hepcidin levels in predicting recurrence and survival outcomes in breast cancer patients. The ROC analysis demonstrated that a cut‐off value of hepcidin > 26.6 ng/mL provided a sensitivity of 80% and a specificity of 66.7% for predicting recurrence, with an AUC of 0.819 (*p* = 0.001), indicating a robust predictive capability of hepcidin levels for disease recurrence in breast cancer patients.

When comparing the AUC for Hepcidin, CRP, and CA 15‐3, this study observed that Hepcidin has superior significance over the other two markers in predicting recurrence in patients. Hepcidin demonstrated a larger area under the curve and higher sensitivity compared to CRP and CA 15‐3 (Table 4).

Kaplan‐Meier survival analysis further supported the prognostic value of serum hepcidin in breast cancer. In the high hepcidin group (hepcidin > 26.6 ng/mL), 12 out of 20 (60%) patients experienced disease recurrence, compared to only 3 out of 19 (15.8%) patients in the low hepcidin group (hepcidin ≤ 26.6 ng/mL). This resulted in a significantly lower progression‐free survival (PFS) in the high hepcidin group (11.6 months and 40%) compared to the low hepcidin group (16.9 months and 84%) (*p* = 0.0027).

Furthermore, the overall survival (OS) analysis demonstrated a significant difference between the two groups. Only two patients (10.5%) in the low hepcidin group died, whereas eight patients (40%) in the high hepcidin group succumbed to the disease. Consequently, the OS mean and rate were higher in the low hepcidin group (17.26 months and 89.5%) compared to the high hepcidin group (14.5 months and 60%) (*p* = 0.032). These results suggest that elevated serum hepcidin levels could potentially serve as a prognostic biomarker for risk of recurrence, PFS, and OS in breast cancer patients.

The observed differences in mortality rates between groups could have influenced our findings, particularly in survival analysis. While the association between high hepcidin levels and increased mortality aligns with prior research, further studies with detailed cause‐of‐death data are necessary to confirm whether hepcidin itself directly impacts survival or whether it serves as a marker of disease severity.

By comparing this results with data from the literature, consistencies and divergences were noted. Several studies have reported the association of elevated serum hepcidin levels with cancer progression and poor clinical outcomes in breast cancer patients [83, 88].

Conversely, other studies have reported different conclusions. One study showed that serum hepcidin levels did not significantly predict recurrence in breast cancer patients, explaining the complexity and variability of the role of hepcidin as a tumor biomarker across different cohorts and study designs [89]. As well, another study demonstrated that although hepcidin is indeed involved in iron metabolism in cancer, its direct impact on survival outcomes might be influenced by other concomitant factors. This suggests the need for more nuanced and multifaceted analyzes [34].

## 5 Conclusion and Limitation

### 5.1 Conclusion

In conclusion, this study contributes to the expanding body of evidence supporting the prognostic significance of serum hepcidin in breast cancer. Elevated levels of hepcidin are notably correlated with heightened rates of recurrence and diminished survival, suggesting its potential as a valuable biomarker for identifying patients at elevated risk of adverse clinical outcomes. Further investigation is warranted to delineate the underlying mechanisms and to validate these observations across larger and more diverse patient cohorts.

Serum hepcidin levels at the time of breast cancer diagnosis may serve as a pivotal predictive indicator of disease severity, prognosis, recurrence, and overall survival, with elevated levels indicative of heightened recurrence risk and diminished overall survival. this findings propose that hepcidin may influence the regulation of tumor growth and metastasis, thereby advocating its potential utility as a biomarker to enhance prognostic assessment and inform therapeutic strategies in breast cancer patients. Future studies should aim to:

➣ Validate hepcidin's role in predicting survival outcomes and guiding treatment decisions.
➣ Assess the clinical utility of hepcidin as a biomarker for disease severity, prognosis, and recurrence.

### 5.2 Limitation

However, this study has certain limitations:

➣ Due to obstacles related to cost and importation of test kits into Syria, the sample size was relatively small, which may have affected the significance of some results. The duration of patient supervision was also relatively short and limited to the duration of master's studies. Large‐scale, long‐term studies would provide more precise results.
➣ We believe that the measurement of other regulators of hepcidin and iron, the most important of which are Interleukin‐6 and bone morphogenetic proteins (BMP 2, 6, 7…), is of unquestionable interest with the aim of understanding the mechanisms by which hepcidin is upregulated in breast cancer contexts. Unfortunately, this objective was not achieved for the same reasons mentioned above.

## Author Contributions

**Zein Al‐Abideen Douba:** writing – original draft, methodology, data curation, formal analysis. **Rama Ibrahim:** supervision.

## Ethics Statement

This study was approved by the Scientific Research Ethics Committee at Tishreen University and Tishreen University Hospital. The ethical approval process was coordinated through the Office of External Relations, led by Dr. Sawsan Ghazal. Written informed consent was obtained from all participants before enrollment. The study was conducted in accordance with the Declaration of Helsinki and all applicable ethical standards for human research.

## Conflicts of Interest

The authors declare that there are no conflicts of interest with respect to the publication of this manuscript. In addition, the authors fully note ethical issues, including plagiarism, informed consent, misconduct, data fabrication and/or falsification, double publication and/or submission, and duplication. The authors declare that there is no financial support or funding for this study. Additionally, there are no conflicts of interest related to this study.

## Transparency Statement

The lead author Zein Al‐Abideen Douba affirms that this manuscript is an honest, accurate, and transparent account of the study being reported; that no important aspects of the study have been omitted; and that any discrepancies from the study as planned (and, if relevant, registered) have been explained.

## Data Availability

The data that support the findings of this study are available from the corresponding author upon reasonable request.
